# Comparison of Anodic and Au-Au Thermocompression Si-Wafer Bonding Methods for High-Pressure Microcooling Devices

**DOI:** 10.3390/mi14071297

**Published:** 2023-06-24

**Authors:** Sylwester Bargiel, Julien Cogan, Samuel Queste, Stefania Oliveri, Ludovic Gauthier-Manuel, Marina Raschetti, Olivier Leroy, Stéphan Beurthey, Mathieu Perrin-Terrin

**Affiliations:** 1 Institut FEMTO-ST, CNRS, Université de Franche-Comté, F-25000 Besançon, France; 2 Aix Marseille University, CNRS/IN2P3, CPPM, Marseille, France; cogan@cppm.in2p3.fr (J.C.);

**Keywords:** bonding technology, anodic bonding, thermocompression bonding, microcooling, microfluidic device, burst pressure test

## Abstract

Silicon-based microchannel technology offers unmatched performance in the cooling of silicon pixel detectors in high-energy physics. Although Si–Si direct bonding, used for the fabrication of cooling plates, also meets the stringent requirements of this application (its high-pressure resistance of ~200 bar, in particular), its use is reported to be a challenging and expensive process. In this study, we evaluated two alternative bonding methods, aiming toward a more cost-effective fabrication process: Si-Glass-Si anodic bonding (AB) with a thin-film glass, and Au-Au thermocompression (TC). The bonding strengths of the two methods were evaluated with destructive pressure burst tests (0–690 bar) on test structures, each made of a 1 × 2 cm^2^ silicon die etched with a tank and an inlet channel and sealed with a plain silicon die using either the AB or TC bonding. The pressure resistance of the structures was measured to be higher for the TC-sealed samples (max. 690 bar) than for the AB samples (max. 530 bar), but less homogeneous. The failure analysis indicated that the AB structure resistance was limited by the adhesion force of the deposited layers. Nevertheless, both the TC and AB methods provided sufficient bond quality to hold the high pressure required for application in high-energy physics pixel detector cooling.

## 1. Introduction

The development of highly integrated microelectronic devices requires ever more efficient thermal management solutions. Efficient cooling systems are also very important to reduce the environmental impact of high-power density electronics cooling [1]. Microfluidic heat sinks have proven to be an excellent solution to meet this challenge [2]. Indeed, the high surface-to-volume ratio in microfluidic channels allows very effective thermal contact between the refrigerant and the heat exchanger. The cooling efficiency can be further improved by reducing the thickness of the channel walls, which in turn reduces the thermal resistance between the heat source and the refrigerant. Since this technology was first introduced in the 1980s [3], numerous developments and optimizations have been made [4]. One of the most notable has been the implementation of flow boiling in microchannels [5], which has allowed for even higher cooling power by using the refrigerant’s latent heat, also ensuring uniform temperature across the structures.

**Figure 1:**
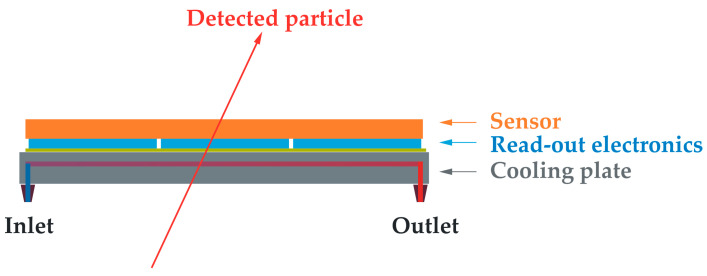
Schematic showing the typical layout of a silicon pixel detector used to localize the crossing point of an impinging charged particle.

Since these seminal works, microchannel cooling technology has found numerous applications in electronics with very demanding power dissipation requirements. This article focuses on the application of this technology to the cooling of silicon pixel detectors used in particle physics experiments. A schematic representing the typical layout of these detectors is shown in Figure 1. A pixelated silicon sensor, typically 100–300 µm thick, is bump-bonded to electronic read-out chips of a similar thickness. The resulting hybrid module has a typical size of a few cm^2^ and is glued onto a plate of matching size, which serves both as a heat sink and a mechanical support.

Although the power dissipation in these detectors remains moderate (1–3 W/cm^2^), the mechanical requirements for such systems are very stringent:

- As the sensor must operate at low temperatures (typically −10 to −30 °C) to mitigate the radiation damage, the coefficient of thermal expansion (CTE) of the cooling system must match that of the sensor;
- As matter perturbs the trajectory of the impinging particles, the cooling plate must be made of a light material and remain as thin as possible (≪1 mm), while maintaining the required mechanical stability, which can be of the order of tens of microns;
- When in-channel evaporative cooling is used, the cooling plate must withstand a very high pressure (up to several hundred bar, depending on the refrigerant chosen).

To fulfil these requirements, the particle physics community has developed silicon microchannel cooling plates. As of today, such devices have been implemented in two detectors: the NA62 GigaTracKer [6] and the upgraded LHCb-VELO (VErtexLOcator) [7]. Their specifications are reported in Table 1. The pressure requirements for the LHCb-VELO upgraded detector have become particularly demanding with the use of biphase CO_2_ as a refrigerant. Although the cooling channels operate at ~14 bar during normal operation at −30 °C, at room temperature (20 °C), the pressure reaches ~57 bar. To establish an operating pressure safety margin in this new technology, the microchannel heat exchanger has been validated to 186 bar. Future high-energy physics applications will face yet higher radiation doses and will probably need to operate at even lower temperatures. Alternative refrigerants have thus been envisaged, including biphase krypton [8], whose pressure exceeds ~100 bar at room temperature. For both the NA62 and LHCb detectors, the cooling plates are fabricated on 8-inch etched silicon-on-insulator (SOI) wafers, bonded together using either hydrophilic or hydrophobic direct bonding (DB) [6,7]. This fabrication process has been reported to be challenging and expensive [7]. The most critical steps were found to be the bonding of large and deeply etched SOI wafers and the soldered attachment of the fluid connectors. Despite these difficulties, Si microchannel cooling offers unmatched lightweight and thermal performance for the vertex detectors that will be required for the next generation of particle physics experiments.

This work aims at improving the fabrication process of microchannel cooling plates in terms of complexity and costs. As alternatives to Si-Si direct bonding (DB), we have investigated two wafer bonding techniques:

- Si-Glass-Si anodic bonding (AB) with a thin-film intermediate borosilicate glass;
- Au-Au thermocompression (TC) bonding.

These two techniques are widespread and have, in principle, less stringent requirements in terms of surface quality and process control, which may translate into higher production yields for the cooling plates. A further reduction in the fabrication cost is expected by using standard Si wafers instead of SOI wafers as well as by decreasing the bonding temperature, which may typically reach 400–700 °C for hydrophobic or 800–1100° for hydrophilic Si-Si DB [9]. Although intermediate layers are involved (glass for AB and gold for TC), they are chemically stable and compatible with the refrigerant fluids expected in the microchannel. Furthermore, the small thickness of these intermediate layers makes the material added to the silicon substrate negligible.

Anodic bonding with thin-film sodium-borosilicate glass, introduced in 1972 for pressure sensors [10], has proven to be an interesting bonding method for microsystem technology. It offers similar bonding performance to standard AB with bulk glass (typ. 0.5–1 mm thick) but with much higher compactness. Due to the thinness of the deposited glass (typ. 2–10 µm), Si-Glass-Si AB can be performed at significantly lower bonding voltage (typ. 50–200 V) and temperature (300–350 °C), introducing lower stress into the bonded system. Nevertheless, the glass deposition process must be optimized to ensure its appropriate composition, thermal and dielectric properties (high electrical breakdown voltage) as well as acceptable surface roughness and residual stress. Si-Glass-Si AB with 2 μm Pyrex 7740 glass (Corning, New York, NY, USA), sputtered on 0.3 μm SiO_2_, was investigated by Hanneborg et al. for sensor applications, demonstrating strong bonding with 400 °C and 200 V applied for 10 min [11]. When compared to sputtering, the e-beam evaporation method—especially plasma-assisted—allows the deposition of glass layers with lower surface roughness (nm range) and lower residual stress, also compatible with the lift-off process [12]. Sassen et al. fabricated a hermetically sealed Si-Glass-Si resonator, bonded at 450–500 °C and 100 V, using an e-beam evaporated 1.5 µm layer (Ra~5 nm) of Schott #8329 glass [13]. Performing AB at 350 °C and 200 V with a 10 µm Schott Borofloat33 (BF33) glass layer deposited with plasma-assisted evaporation, Conti et al. demonstrated a Si-Glass-Si microfluidic flow control valve operating at high pressure up to 20 bar [14].

TC bonding is considered a versatile, low-cost and industrially attractive method compatible with a wide variety of thin-film metal layers and different deposition methods, including magnetron sputtering, e-beam evaporation and electrodeposition. Among the various TC approaches, bonding with gold (Au-Au) is the most popular due to its advantageous plastic deformation and high chemical stability (oxidation resistance), resulting in less demanding process requirements. Since Au creates a eutectic structure with Si at ≥363 °C, gold TC bonding on Si substrates is typically carried out at lower temperatures (250–350 °C) and may involve additional barrier layers (NiCr, TiW, Pt) to prevent the thermodiffusion of gold. Tsau et al. achieved a strong bond between e-beam evaporated Ti/Au (100/800 nm) layers, applying 0.5 MPa pressure for 10 min at 300 °C [15]. Goorsky et al. reported that hermetic Au-Au bonds could be achieved at 200 °C under 3 MPa pressure for 15 min, using sputter-deposited TiW/Au (400/1200 nm) films with intermediate values of initial surface roughness (3–5 nm) [16]. Hermetic Au-Au bonding for wafer-level MEMS packaging was also reported by Charlot et al. [17]: the TC process was carried out at 420 °C and 5.7 MPa using electroplated 3000 nm Au on evaporated TiW (50 nm) diffusion barrier and Ti/Au (50/500 nm) seed layers.

In this work, we report on a comparison of the pressure resistance of microfluidic test structures fabricated using AB and TC bonding methods. The main goal is to verify the compatibility of these bonding methods with microcooling applications where a very high-pressure resistance (≥200 bar) is required. The bonding was performed on four-inch Si wafers, using evaporated BF33 glass (AB) and sputtered Ti/Au (TC) as intermediate layers. This TC metal configuration was selected to be simple and easily accessible. The two tested bonding methods are found to offer sufficient bond quality to hold the high pressure required for the application of particle physics detectors. In addition, these bonding techniques could potentially be used to bond connectors to the cooling plates.

## 2. Materials and Methods

The bonding strength was evaluated, as in [7], through a series of destructive pressure burst tests. The maximum internal pressure reached in microfluidic test structures before breakage was measured. Fabrication began with the etching of a series of microfluidic structures on a four-inch-diameter silicon wafer. The etched wafer was then bonded to a second silicon wafer to close the structures. The silicon wafer sandwich was diced into (20 × 10 mm^2^) dies, containing a single test structure. The chip fabrication processes and the pressure test setup are described in the following sections.

### 2.1. Design of Test Structures 

**Figure 2:**
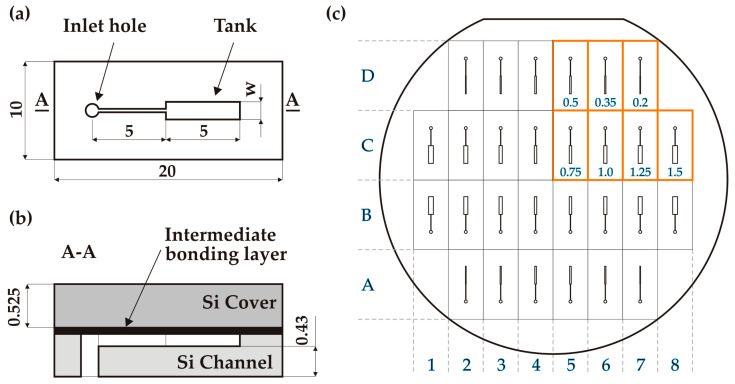
Design of the pressure test structures: (**a**) plan view of the channel base; (**b**) side view of an individual chip after bonding; (**c**) wafer-level design of the channel structures with tank widths (mm) die naming convention (A1, B1, …, D8).

The microfluidic test structures consisted of an upper flat cover plate bonded to a lower micromachined channel base, via an intermediate bonding layer (glass or gold depending on the bonding method), as shown in Figure 2. Each channel base contained a single rectangular “tank” fed from a (0.8 mm diameter) inlet hole through a 50 µm straight channel. The tank and channel were both 5 mm long and 70 µm deep. The channel base and cover plate were 525 µm thick, so the minimal wall thickness (at the bottom of the tank) was ~455 µm. At the wafer level, 28 test structures were designed with seven different tank widths (w = 0.2/0.35/0.5/0.75/1.0/1.25/1.5 mm), as shown in Figure 2. The total wafer-to-wafer bonding surface area was 76.6 cm^2^.

### 2.2. Fabrication and Characterization of Test Wafers

#### 2.2.1. Cover Wafer for Anodic Bonding Studies

The cover wafer used in the AB process is a 4-inch-diameter, 525 µm-thick silicon wafer (p-type) with a SiO_2_/BF33 glass layer deposited on one side. The 4 µm-thick BF33 layer was deposited by plasma-assisted evaporation on the Si wafer coated with 1 μm SiO_2_. The wafer was purchased pre-coated from MSG Lithoglas GmbH (Dresden, Germany) and was characterized in terms of its 3D surface profile (Verifire^TM^ GPI XP interferometer, Zygo Corp, Middlefield, CT, USA) and glass surface roughness (Bruker Dektak XTA profilometer, Billerica, MA, USA). The glass layer uniformity was measured with an optical reflectometer (F50-EXR, Filmetrix Inc., San Diego, CA, USA) in the wavelength range 380–1700 nm. The wafer curvature was also measured as a function of temperature with a thin-film stress measurement system (500TC, FSM, Milpitas, CA, USA) to verify if the initial residual stress of the deposited thin films could be released by annealing before or during the bonding process. The temperature profile had two steps: heating from 20 °C to 400 °C (5 °C/min), followed by natural cooling back to 20 °C with continuous measurement of the wafer profile evolution.

#### 2.2.2. Cover Wafer for Thermocompression Bonding

The cover wafer is a double-side polished (DSP), 525 μm-thick, 4-inch and n-type (100) silicon wafer with a total thickness variation (TTV) of less than 1 µm. This low TTV is chosen to fulfil the more stringent requirements of TC bonding compared to anodic bonding. The cover wafer was selected among a few candidates to match the 3D surface profile of the channel base.

#### 2.2.3. Channel Wafers

**Figure 3:**
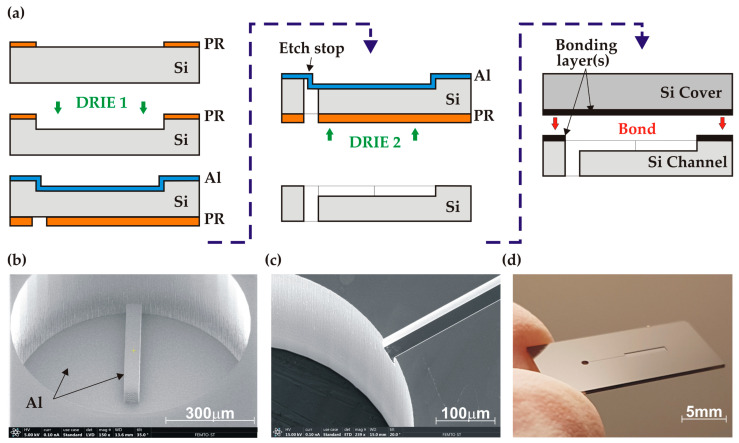
Fabrication of the test structure: (**a**) simplified process sequence; (**b**) SEM image of the inlet hole etching (DRIE 2) with the exposed Al stop layer; (**c**) SEM image of inlet hole and channel after Al removal; (**d**) Si channel base (2 × 1 cm^2^) following wafer dicing.

The channel wafers were prepared on 525 µm-thick, 4-inch and n-type (100) DSP silicon substrates with a resistivity of 1–10 Ω·cm and TTV < 3 µm. Two successive deep reactive ion etching (DRIE) processes were performed using a Rapier process module (SPTS Technologies Ltd., Newport, UK), as shown in Figure 3a. For the first etching of channels (DRIE1), a 1.2 µm-thick layer of S1813 Microposit photoresist (PR) from Shipley (Marlborough, MA, USA) was spin-coated onto the wafer front side with an ACS200 coater (Suss Microtec, Garching, Germany) and photolithographically patterned using an EVG601 aligner (EVG, St. Florian am Inn, Austria). The channels were then etched to a depth of 70 ± 2.5 µm. For the second etching of inlet holes (DRIE2), a 0.6 µm-thick aluminium layer was first magnetron sputtered (MP700, Plassys, Marolles-en-Hurepoix, France) on the already etched wafer front side in order to create an etch stop layer. Next, a 6 µm-thick AZ10XT photoresist (Microchemicals, Ulm, Germany) was spin-coated and patterned on the back side of the wafer, followed by the DRIE etching through the wafer until exposing the Al etch stop (Figure 3b). The average etch rate was 10 µm/min and the Si etch selectivity over PR was 150:1. After the DRIE etching, the photoresist mask was stripped in acetone. The wafer next underwent O_2_ plasma cleaning (Gigabatch 360M, PVA TePla AG, Wettenberg, Germany) and the aluminium layer was removed using a commercial Al etch solution at 40 °C. The channel depth as well as the depth uniformity across the wafer were measured using a Dektak profilometer, and inspections were performed using a Thermoscientific Apreo S (Thermo Fisher Scientific, Waltham, MA, USA) scanning electron microscope (SEM). The homogeneity of the etching depth (target 70 µm) was (70.3 ± 0.2) µm. The obtained channel structure is shown in Figure 3c,d.

### 2.3. Bonding Procedure

#### 2.3.1. Wafer Preparation

Before bonding, the wafers were cleaned in acetone and ethanol to remove dust and organic contaminants. The cover wafers (for both AB and TC) were wet cleaned in a “Piranha solution” (33% H_2_O_2_: 67% H_2_SO_4_) and rinsed in deionised (DI) water. Due to the relatively deep structuration of the channel wafers and potential problems rinsing away the viscous Piranha solution with DI water, they were dry cleaned by O_2_ plasma in the TePla microwave reactor (parameters 300 W, pressure 0.8 mbar, O_2_ flow 50 sccm). The cleaned wafer pairs were either used directly for AB or underwent the Ti/Au thin-film deposition procedure for TC bonding. 

#### 2.3.2. Bonding Equipment

All bonding operations were performed in an AWB04 bonder (AML Ltd., Oxfordshire, UK), which allows the integration into a standard bonding recipe of several in situ operations, including optical alignments, surface O_2_ plasma treatments or DI water vapour injections. In the AB configuration, graphite and tungsten platens were installed as upper (cathode) and lower (anode) platens, respectively. Specific wafer side-clamping on the upper platen avoided any contact between the bonding surfaces and the bonder parts (flags, jigs, etc.). The wafer alignment was made either at room temperature (TC) or at bonding temperature (AB). 

#### 2.3.3. Anodic Bonding Process

To achieve stable bonding without electrical breakdown in the very thin glass layer, a voltage in the range 30–60 V and a temperature around 300 °C are, in principle, sufficient [12]. Nevertheless, in this work, the voltage value was increased to 180 V due to the thickness of the oxide layer (1 μm) in the bonding stack.

**Figure 4:**
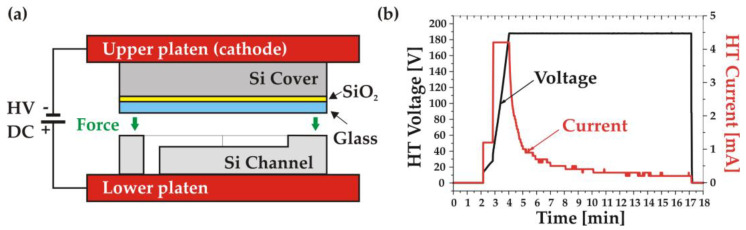
Anodic bonding: (**a**) schematic of the bonding setup; (**b**) voltage and current measured during the bonding process.

The process started with surface activation using radical activation (RAD) with O_2_ plasma, which was carried out within the vacuum chamber of the bonder in the configuration illustrated in Figure 4a. The activation was performed over 10 min with an RAD voltage and current of 600 V and 100 mA. The activated surface was then treated in situ with DI water vapour to complete the hydrophilization procedure. To relax the residual stress, the cover wafer underwent a short annealing in vacuum at 400 °C for 20 min while keeping the channel wafer at 150 °C. Once the temperature of the two wafers was stabilized at the desired bonding temperature (350 °C), they were aligned and contacted with a bonding force of 500 N. The AB process was completed by increasing the DC voltage progressively up to 180 V, keeping the bonding current limited to 4 mA, as shown in Figure 4b. This method was intended to ensure the uniformity of the bonding current, reducing the stress level in the final bonded stack. The bonding process current was maintained for 15 min with a total transferred charge of around 750 mC (Figure 4b). 

#### 2.3.4. Au-Au Thermocompression Bonding

The adhesion (Ti) and bonding (Au) layers were deposited immediately after the channel and cover wafers had been cleaned. The deposition was made using the MP700 magnetron sputtering system. The process started with the evacuation of the chamber to a background pressure of 10^−5^ Pa. The distance between the planar cathodes and the substrate was then set to 10 mm and the surfaces were prepared for the subsequent thin-film depositions with an Ar reactive plasma. Finally, the layers were deposited at a working pressure of 0.9 Pa: a 20 nm-thick adhesion layer with a target current of 1 A first, followed by the 350 nm-thick Au bonding layer with a target current of 0.3 A. A thickness non-uniformity of ±11% was obtained with a tensile stress of 110 MPa. The average roughness (Ra) of the Au surface was 1.09 nm.

**Figure 5:**
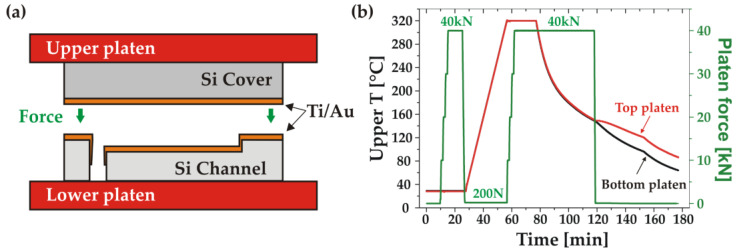
Thermocompression Au-Au bonding: (**a**) schematic of the bonding setup; (**b**) measured temperature of the upper (red) and lower (black) platens and applied force (green) during the bonding process.

After the Ti and Au depositions, the wafer pair was installed in the bonder and the chamber was evacuated to 7.0 × 10^−7^ Pa. After alignment, a pre-bonding step was performed at room temperature under a piston force of 40 kN (5.22 MPa) for 5 min. Next, the wafer stack temperature was ramped up while keeping a small, controlled force of 200 N, followed by a final bonding step at a temperature of 320 °C and under a force of 40 kN for 20 min. The bonded stack was held under pressure during a controlled cool down to 150 °C. The registered temperature and force characteristics of the bonding process are shown in Figure 5.

### 2.4. Acoustic Imaging Analysis

**Figure 6:**
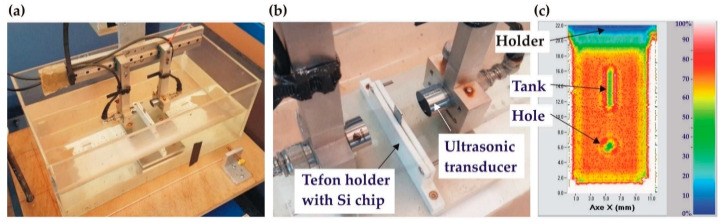
Scanning acoustic microscopy (SAM) measurements of Au-Au bonded wafers: (**a**) home-built SAM system, zoom of the water tank showing the emitter/receiver and sample holder; (**b**) zoom of the sample holder; (**c**) typical SAM image of a well-bonded test structure.

Scanning acoustic microscopy (SAM) in transmission mode was used to visualize possible defects at the Au-Au bonding interface in the wafer stack as well as in the individual test structures post-dicing. A home-built SAM system equipped with two focussed acoustic transducers (Sonaxis, Besançon, France) and an emitter/receiver controller (Sofranel, Sartrouville, France) was integrated into an X–Y–Z translation stage. The transducers have an active diameter of 19 mm and a focal length of 30 mm, and they operate at a central frequency of 15 MHz, providing an imaging resolution close to 200 µm (−6 dB). Figure 6a,b shows the water reservoir with the test sample clamped in the Teflon holder. In the calibration procedure, the attenuation was adjusted such that the highest impedance signal on the sample corresponded to 70% of the full scale. As a result, the area with the highest acoustic transmission in the tested sample (best bonding quality) is represented by red/orange in SAM images, whereas bonding defects (voids) or air-filled channels larger than 200 µm are clearly visible as green/blue areas. An SAM image of a well-bonded test structure is shown in Figure 6c.

### 2.5. Wafer Dicing

The bonded wafer stacks were diced using a DAD321 dicing saw (DISCO, Tokyo, Japan), operating with a standard 100 µm blade.

### 2.6. Pressure Resistance Test Bench

**Figure 7:**
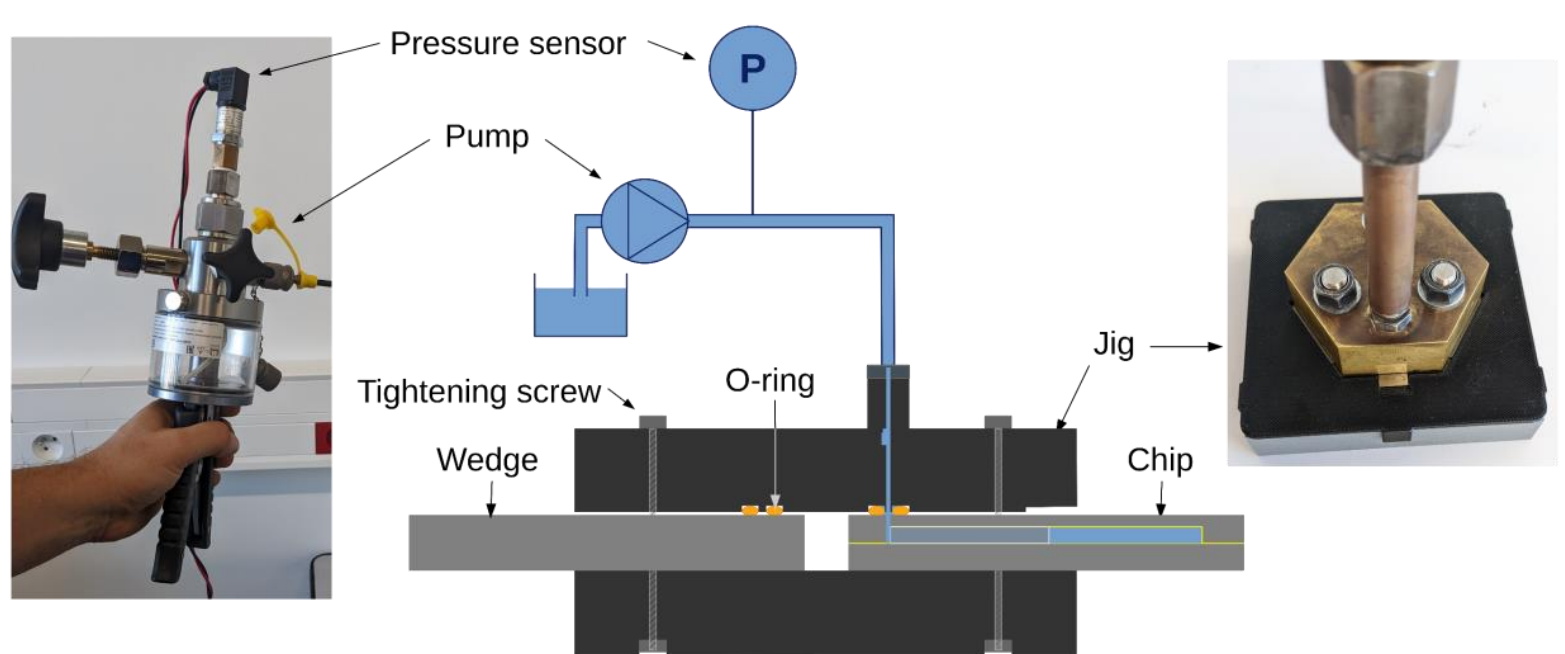
Schematic of the high-pressure test bench for destructive burst pressure characterisation of bonded die test structures.

A dedicated test bench was built to measure the pressure resistance of the bonded test structures (Figure 7) using a DI water. The test bench was based on a CPP700-H hand-operated hydraulic pump (Wika, Herblay, France), capable of generating pressures up to 700 bar, and instrumented with a Gems/3100 pressure sensor (Gems Sensors, Plainville, CT, USA). The 4–20 mA analog pressure signal is converted to a voltage and digitized with a 12-bit ADC (Microchip MCP3208, Chandler, AZ, USA) read via the SPI bus of a micro-computer (Raspberry Pi3 model B, Cambridge, UK). Measurements are recorded at a rate of ~3 Hz using custom Python-based data acquisition software. An in-house manufactured jig allows the connection of the pump outlet to the inlets of the test structures. The (700 bar) sealing is ensured by an O-ring with a 2 mm inner diameter and 1.6 mm cross-section inserted in a groove with a 7 mm external diameter.

**Figure 8:**
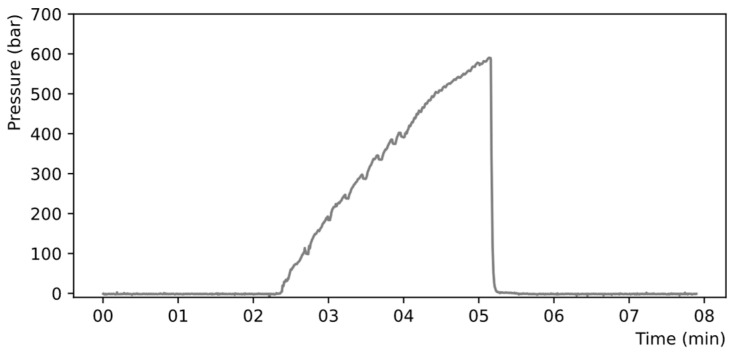
Typical rise of pressure inside a chip recorded with the high-pressure bench.

The pressure inside the microstructure was raised at a typical rate of 200 bar/minute until the failure of the chip—identified as a sudden pressure drop—or until the maximum attainable pressure of 690 bar of the pump was reached. A typical time-dependant pressure ramp is shown in Figure 8. The uncertainty on the burst pressure measurement is estimated to be around ±20 bar due to the time capture precision of the data acquisition system.

### 2.7. Analysis after High-Pressure Destructive Test

Broken samples with exposed bonding interfaces were investigated by optical microscopy to identify the failure mode. Surface profile measurements were performed using a mechanical profilometer after sample wet cleaning (Acetone/Ethanol/Piranha). Additionally, qualitative chemical analyses of bonding interfaces were made using a high-resolution energy dispersive spectrometry detector (Octane Elect Plus, EDAX, Pleasanton, CA, USA), integrated within the SEM device. SAM imaging was also performed on samples that preserved their integrity after the pressure test.

## 3. Results

### 3.1. Anodic Bonding Results

#### 3.1.1. Thin-Film BF33 Glass Characterization

The cover wafer was characterized to verify its compatibility with the anodic bonding process in terms of the roughness of deposited glass and overall wafer geometry. According to the general guidelines for the AB process, the preferred surface roughness is around Ra = 1 nm, while the maximum permissible roughness should not exceed Ra = 10 nm with a maximum peak-to-valley of 100 nm. In addition, the critical bow/warp of standard 500 µm-thick, 4-inch wafers is in the order of 40 µm.

**Figure 9:**
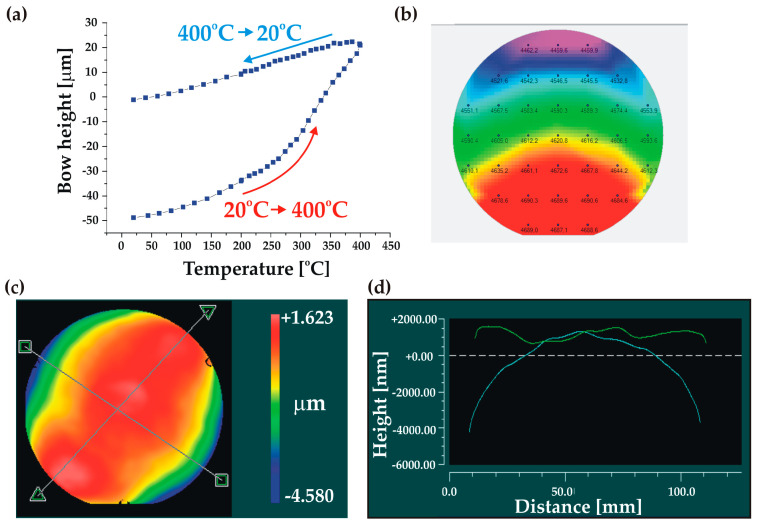
Characterization of the Si cover wafer with SiO2/BF33 intermediate layers for bonding: (**a**) wafer bow measurement showing the stress relaxation during annealing; (**b**) thickness uniformity of BF33 layer; (**c**,**d**) 3D and 2D wafer profiles after annealing, respectively.

The roughness measurement revealed a very smooth BF33 glass surface with some relatively high-amplitude local defects. The roughness measured in three different places on the wafer was in the range Ra = 0.44–1.74 nm, whereas the maximal total height of measured profiles was Rt = 62 nm. The uniformity of the deposited layer was very good (2.5–2.8%). The wafer profile analysis showed a significant initial bow of P-V = –48.8 µm at room temperature, indicating the presence of residual stress in the SiO_2_/BF33 layers. However, the annealing process, following a thermal cycle of 20 °C –> 400 °C –> 20 °C (Figure 9a), allowed an almost full relaxation of the stress in the cover wafer and a significant reduction in the wafer bow to P-V = −1.2 µm (Figure 9c,d). After the thermal treatment, the cover wafer fulfilled the AB requirements.

#### 3.1.2. Bonding Process

**Figure 10:**
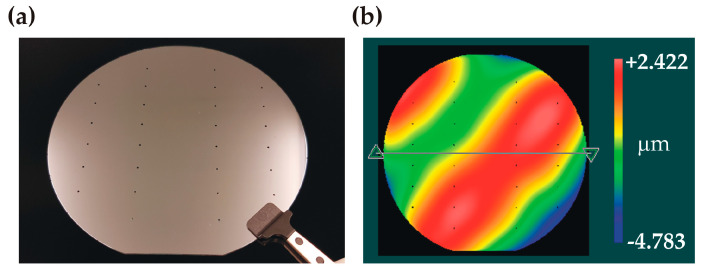
Characterization of the anodically bonded wafer stack: (**a**) photo of the bonded wafer stack (channel side, visible inlet holes); (**b**) 3D profile showing the wavy surface.

No electrical breakdown was observed during the bonding process, indicating good film quality. The bonded wafer pair is shown in Figure 10a. Infrared inspection of the bonded pair showed a uniform bonding interface without voids. The bonded stack had a wavy surface profile with a small value of P-V = 7.2 µm (Figure 10b). The stack was diced into individual test samples with a 100% yield, demonstrating that the overall bonding was strong enough to withstand the force exerted by the dicing blade. Individual samples underwent the high-pressure test procedure.

#### 3.1.3. Pressure Resistance Tests

**Figure 11:**
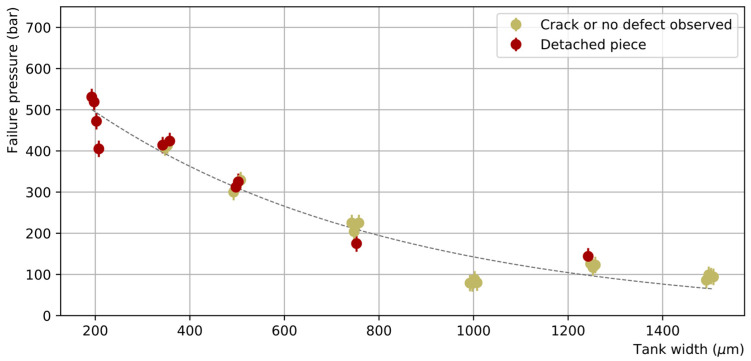
Failure pressure of the AB test samples as a function of the channel tank width (the points have been slightly shifted horizontally around the true tank width value to avoid overlapping). Two categories of failure are distinguished: failures where a large piece is detached, showing clear unsealing over a large area (crimson), or failures where only a thin crack on the surface or no defect at all is observed (khaki). To guide the eye, a decreasing exponential function was empirically fitted to the data points.

The measured failure pressures as a function of the channel tank width are shown in Figure 11. In general, AB samples demonstrated high resistance to hydraulic pressure, reaching up to 100–530 bar before breaking depending on the tank width. As the moment of the force induced by the pressure on the edge of the tank increases with the tank width, the smaller the tank width, the higher the failure pressure. All dies with a tank width smaller than 750 µm, representing about 40% of the full sample, sustained pressures higher than 250 bar. Good reproducibility of the failure pressure was also observed (four samples were measured for each tank width) with a maximum dispersion (rms) of 10% of the mean.

#### 3.1.4. Post-Pressure Test Analysis

**Figure 12:**
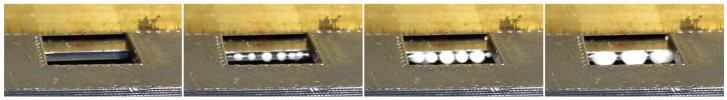
Stop-motion pictures showing the DI water leaking from the bonding interface of the tested die (one image every 1/20 s). Several leaking sources are observed simultaneously.

Two kinds of die failure were observed: either the water leaked from the bonding interface, as illustrated in Figure 12, or a large piece of either the cover or the channel base was detached. The occurrence of these failure types is given in Table 2 for each tank width. Leaks at the interface were mostly observed for large tank widths while pieces were detached at small tank widths, thus at higher pressures. 

##### First Failure Type: Detached Piece

**Figure 13:**
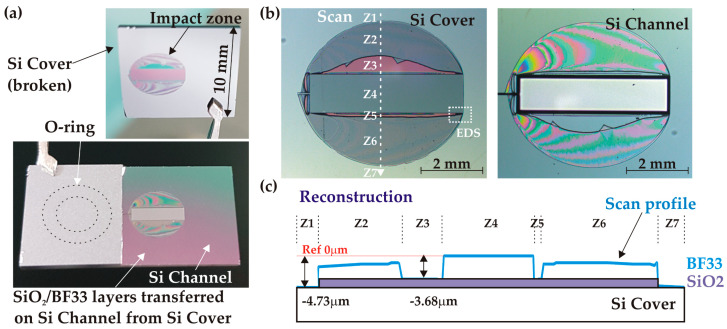
Analysis of a broken AB sample (C2, w = 1.25 mm) after high-pressure test: (**a**) general view; (**b**) impact zone (IZ) both on cover and channel parts; (**c**) profile of the IZ on cover.

The detached piece always extends over the full die width (1 cm) while preserving the chip integrity in the area around the O-ring sealing the inlet. A typical example of such a failure is shown in Figure 13a. The exposed inner parts show the channel tank surrounded by a symmetrical round-shaped “impact zone” (IZ), indicating that the bonding interface generally opened up at a critical failure pressure. Seven areas (Z1-Z7), illustrated in Figure 13b, can be identified in the IZ. A more detailed analysis of these areas has been performed based on optical microscope observations and mechanical profilometer measurements. Considering the IZ on the detached cover part from the centre to the periphery, we observe the unaffected part facing the tank (Z4), a local delamination of the glass in zones Z3 and Z5 exposing the pink-coloured SiO_2_ layer, and finally, a flat breakage in the core of the glass layer (Z2, Z6). The IZ is surrounded by a flat grey area (Z1, Z7) identified as silicon. Taking this area as a reference, profile measurements show that the central part Z4 represents, as expected, the full initial thickness of SiO_2_/BF33 layers (4.73 μm) whereas the Z3 area corresponds only to the thickness of the SiO_2_ (1.05 μm). This indicates that the BF33 layer located directly at the tank edge was detached from the SiO_2_ layer, remaining attached instead to the tank base wafer. Outside the IZ, the whole double layer of SiO_2_/BF33 was transferred onto the Si channel base (Figure 13a, lower vignette), indicating the delamination of the Si/SiO_2_ interface. The reconstructed profile is shown in Figure 13c. 

**Figure 14:**
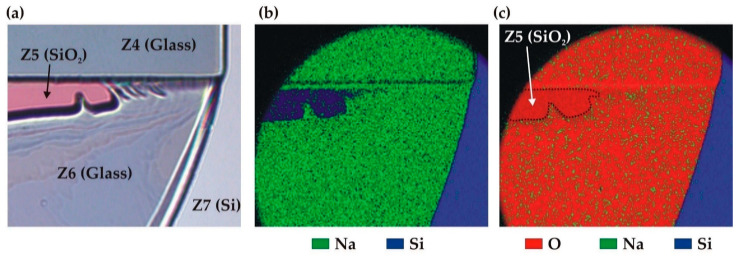
EDS analysis of the broken bonding interface of the C2 sample: (**a**) zoom on the EDS area inside the IZ (optical microscope); (**b**) map of Na and Si elements; (**c**) map of O, Na, and Si elements.

A qualitative analysis of the IZ using energy dispersive spectrometry (EDS) in the restricted area indicated in Figure 13b confirmed the above-mentioned material identification. Figure 14 illustrates the map of three elements (O, Na, and Si) found in this zone (the dominant element is shown in each pixel). The BF33 glass can be identified in the Z4 and Z6 zones by the simultaneous presence of the Si and O elements coming from the SiO_2_ component (81% of the glass composition), and the Na element from Na_2_O (4%). Similarly, the SiO_2_ layer in the Z5 zone is confirmed by the exclusive presence of the Si and O elements, whereas the silicon surface in Z7 is identified by the predominant presence of the Si element in all measured cases.

##### Second Failure Type: Leak at the Interface

**Figure 15:**
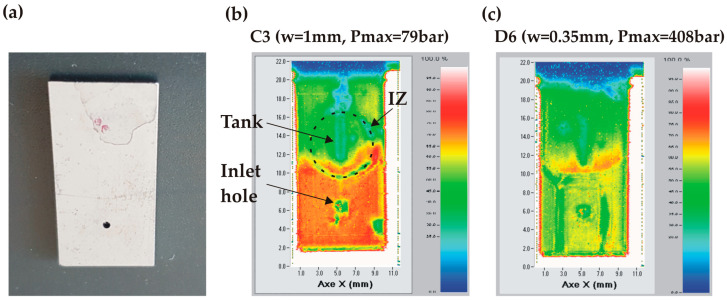
SAM images of AB dies having leaked at the bonding interface: (**a**) non-broken die after high-pressure test; (**b**) SAM image of chip C3 (w = 1 mm) that failed at a relatively low pressure (79 bar); (**c**) SAM image of chip D6 (w = 0.35 mm) having failed at high pressure (408 bar).

The dies where the water leakage was observed at the bonding interface show either a thin crack extending over the full die width or no apparent defect at all. A typical example of a die without an apparent defect is shown in Figure 15a. The SAM analysis of a chip failing at lower pressure (<100 bar) revealed that the unsealing occurred on half of its surface around the tank (green area) while still preserving the die integrity in the area around the inlet due to the clamp ensuring the tight fluidic connection (Figure 15b). Another example in Figure 15c shows a failure at much higher pressure (408 bar) resulting in an almost complete disconnection of the bonded structure. 

Hence, we conclude that the maximum pressure that can be sustained by the AB dies is limited by the adhesion forces of the deposited BF33/SiO_2_ layers rather than by the much stronger anodic bonding at the BF33/Si interface.

### 3.2. Thermocompression Bonding Results

#### 3.2.1. Bonding Process

**Figure 16:**
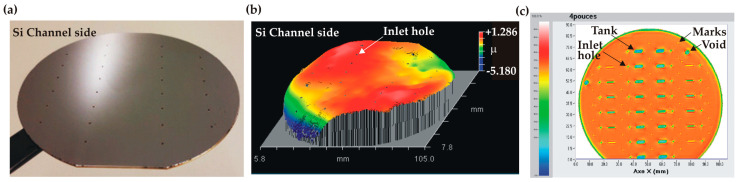
TC bonding results: (**a**) picture of the bonded wafer stack (channel side uppermost); (**b**) 3D surface profile (channel side); (**c**) SAM imaging of the wafer stack (the bottom part is hidden by the Teflon holder).

The bonded wafer stack is shown together with SAM imaging in Figure 16. Except for two voids without particular significance—located at the border—a uniform acoustic transmission was observed over the whole bonding interface. A flat surface profile was also measured with a small height of P-V = 6.4 μm. 

#### 3.2.2. Pressure Resistance Tests

**Figure 17:**
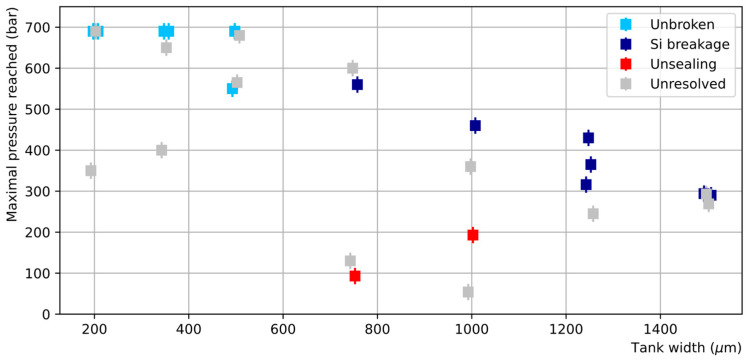
Maximal pressure reached in dies bonded by TC as a function of the channel tank width (the points have been slightly shifted horizontally around the true tank width value to avoid overlapping). Four categories are distinguished: dies reaching the indicated pressure without breaking (light blue), dies showing clear signs of breakage in the silicon only (dark blue), dies showing evidence for unsealing (red), and dies for which no diagnostic was possible (light grey).

The failure pressures of dies bonded by the TC process are shown in Figure 17. The bond resistance is generally very good with more than 80% of the dies sustaining pressures exceeding 250 bar, including those with the largest tank widths. In particular, five dies reached the maximum attainable pressure of 690 bar without breakage. Nevertheless, significant dispersion of the obtained failure pressures is observed, with some failures appearing at unexpectedly low pressures.

**Figure 18:**
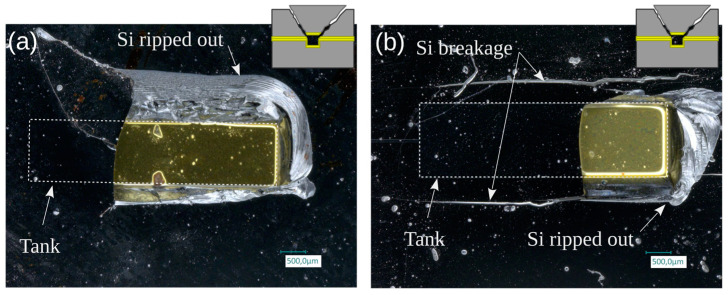
Micrographs of broken samples showing clear breakage of the silicon on top of the tank base: (**a**) failure pressure: 316 bar (wafer position C2, w = 1.25 mm); (**b**) failure pressure: 293 bar (wafer position C1, w = 1.5 mm).

Visual inspection clearly showed that in most cases the breakages occurred in the silicon, either in the channel or cover side, with no sign of unsealing, leading to fracture of the Si parts at high failure pressures. Two examples of large-tank-width dies with failure pressures exceeding 300 bar are shown in Figure 18. At such high pressures, the simultaneous action of bending and shear stresses exceeds the critical stress that Si material can withstand [18].

**Figure 19:**
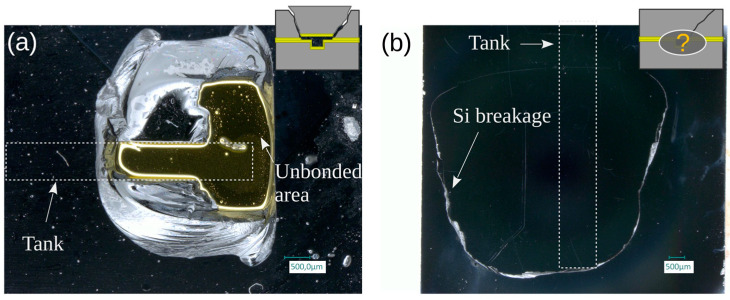
Micrographs of TC-bonded die pair that failed at unexpectedly low pressure. The tank width is 750 µm in both cases: (**a**) die showing clear signs of unbonding (wafer position B4, failure pressure: 93 bar); (**b**) die with an ambiguous failure type as the broken part is not fully detached (wafer position C4, failure pressure: 130 bar).

In addition, however, there were a few outliers that failed at relatively low pressures. One of these, illustrated in Figure 19, shows clear signs of unbonding—revealing the Au layer surrounding the tank—possibly linked to a local defect at the tank edge, while others present only cracks on their surface and have failure modes that are harder to interpret.

**Figure 20:**
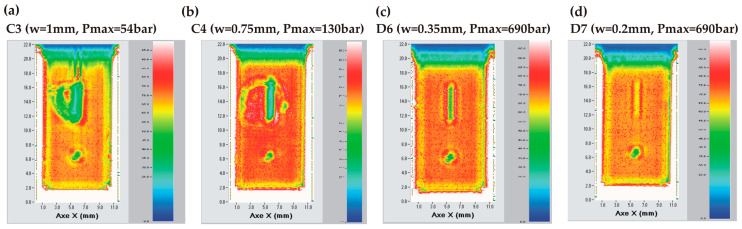
SAM images of TC bonded dies after high-pressure test: (**a**) die (wafer position C3; w = 1 mm) with a crack and having leaked at relatively low pressure (54 bar); (**b**) die (wafer position C4; w = 0.75 mm) with a crack and having failed at 130 bar; (**c**) non-broken die (wafer position D6; w = 0.35 mm); (**d**) non-broken die (wafer position D7; w = 0.2 mm).

SAM analyses have been performed on dies showing such a crack (Figure 20a,b) or surviving the high-pressure test without breaking (Figure 20c,d). The SAM images of the dies presenting a crack reveal that the dies were damaged around the tank. The unbroken dies show that their integrity is preserved over the full surface. Hence, we conclude that the maximum pressure that can be sustained by a TC die may be limited by the silicon strength itself if a sufficient bonding quality is ensured through the exclusion of local defects. 

## 4. Discussion

The obtained results show several advantages of the Si-Glass-Si AB, including a good pressure resistance (tested samples with w ≤ 500 μm sustained at least 250 bar) and very good reproducibility of failure pressures. Moreover, the AB process generates little stress in the bonded wafer stack. However, even if AB may be performed with standard bonding equipment, access to this technique is limited. The plasma-assisted evaporation of a BF33 layer is not a common method in cleanroom facilities. 

TC bonding can also be considered a very appealing technique as it is widespread and available in most cleanroom facilities. The maximal pressure values reached by the TC samples generally surpass those of the AB samples, the limitation in the TC case generally being set by the Si strength. As TC bonding is also a simpler process in terms of substrate preparation and process control than AB, it appears to be the more promising technology. However, the reproducibility of the results is not as good as for the AB. 

Two factors appear to be responsible for the variability in TC bond resistance: defects in the case of a few outliers leading to fracture at unexpectedly low pressures, together with the intrinsic dispersion due to the breaking mechanism in Si. By contrast, the delamination of the deposited layers in the AB samples seems to occur at a critical pressure value, leading to lower dispersion of the failure pressures. 

The TC outliers, such as that shown in Figure 19a, are probably due to local defects on the bonding interface such as a hard particle or other local variations affecting the bonding quality. From this perspective, further optimizations of the proposed TC process are necessary, including the reduction in the particulate contamination, the improvement of the metal thickness uniformity (currently ±11%), and the bonding pressure uniformity. The bonding strength may also be affected by the thermal inter-diffusion between the deposited metal layers and Si substrate at 320 °C, which could be avoided by the use of an additional diffusion barrier layer.

Since only one wafer stack was processed for each bonding method, no firm conclusions can yet be drawn on stack-to-stack repeatability. However, since both methods employ only standard and well-controlled processes, no adverse repeatability issues are expected. A comparison of the maximum failure pressures reached with the AB and TC methods reported in this work with those obtained with DB in [7] is presented in Table 3. Although the Si is thinner in the DB case—making a direct comparison with this work difficult—this comparison suggests that TC bonding could be as good as DB. Indeed, the maximum failure pressure is limited by the Si strength in both cases.

## 5. Conclusions

This work aimed at reducing the complexity and costs of the fabrication process for microfluidic Si cooling devices operating at high pressures in particle physics experiments. Two bonding methods were investigated and successfully tested on four-inch wafers. The anodic bonding of a structured Si wafer to a Si wafer coated with an evaporated 3.7 μm BF33 glass thin film was made under the conditions of a standard bonding temperature (350 °C) and low voltage (180 V). Au-Au thermocompression bonding with sputter-deposited Ti/Au layers (20 nm/350 nm) was carried out at a temperature of 320 °C and a bonding pressure of 5.22 MPa. The results of these pressure resistance experiments are encouraging since high hydraulic pressures were achieved for both types of bonding, reaching a maximum of 530 bar for AB and 690 bar for TC samples. From this perspective, both the TC and AB methods could provide sufficient bond quality for microcooling applications in high-energy physics, including the most demanding ones based on bi-phase krypton cooling. Of the two, however, currently, TC bonding seems to be the more promising technology in terms of industrialization (lower cost, faster processing), but additional optimizations are required to improve the bonding uniformity.

## Figures and Tables

**Table 1:** Specification of the cooling system of the two particle physics detectors implementing Si microchannel cooling [6,7].

Detector Name	Dimensions (cm²)	Total Plate Thickness (µm)	Refrigerant Type	Operating Pressure (bar)	Operating Temp. (°C)
NA62 GigaTracKer	7 × 8	210	Liquid C_6_F_14_	3	[−10; 20]
Upgraded LHCb-VELO	11.37 × 11.65	500	Bi-phase CO_2_	14–57	[−30; 20]

**Table 2:** Number of AB dies with failure type identified, either as a detached piece or with a leak at the interface.

Tank width (µm)	200	350	500	750	1000	1250	1500
Detached piece	4	2	2	1	-	1	-
Leak at interface	-	2	2	3	4	3	4

**Table 3:** Comparison of the maximum failure pressures reached in various tank width and silicon wafer thickness configurations.

Bonding Type	Tank Width	Min. Si Thickness	Max. Failure Pressure	Failure Type	Ref.
DB	200 µm	140 µm	>450 bar	No failure	[7]
AB	200 µm	430 µm	530 bar	Interface	This work
TC	200 µm	430 µm	>690 bar	No failure	This work
DB	500 µm	140 µm	280 bar	Si breakage	[7]
AB	500 µm	430 µm	330 bar	Interface	This work
TC	500 µm	430 µm	>690 bar	No failure	This work
DB	1250 µm	140 µm	80 bar	Si breakage	[7]
AB	1250 µm	430 µm	125 bar	Interface	This work
TC	1250 µm	430 µm	430 bar	Si breakage	This work

## Data Availability

The data presented in this study are available on request from the corresponding author.

## References

[1] Van Erp R., Soleimanzadeh R., Nela L., Kampitsis G., Matioli E. (2020). Co-Designing Electronics with Microfluidics for More Sustainable Cooling. Nature.

[2] Lee D.-Y., Vafai K. (1999). Comparative Analysis of Jet Impingement and Microchannel Cooling for High Heat Flux Applications. Int. J. Heat Mass Transf..

[3] Tuckerman D.B., Pease R.F.W. (1981). High-Performance Heat Sinking for VLSI. IEEE Electron Device Lett..

[4] Kandlikar S.G. History, Advances, and Challenges in Liquid Flow and Flow Boiling Heat Transfer in Microchannels: A Critical Review. Proceedings of the 2010 14th International Heat Transfer Conference.

[5] Moriyama K., Inoue A., Ohira H. (1993). The Thermohydraulic Characteristics of Two-Phase Flow in Extremely Narrow Channels: The Frictional Pressure Drop and Void Fraction of Adiabatic Two-Component Two-Phase Flow. Heat Transf.—Jpn. Res..

[6] Rinella G.A., Feito D.A., Arcidiacono R., Biino C., Bonacini S., Ceccucci A., Chiozzi S., Gil E.C., Ramusino A.C., Danielsson H. (2019). The NA62 GigaTracKer: A Low Mass High Intensity Beam 4D Tracker with 65 Ps Time Resolution on Tracks. J. Instrum..

[7] De Aguiar Francisco O.A., Byczynski W., Akiba K., Bertella C., Bitadze A., Brock M., Bulat B., Button G., Buytaert J., De Capua S. (2022). Microchannel Cooling for the LHCb VELO Upgrade I. Nucl. Instrum. Methods Phys. Res. Sect. A Accel. Spectrometers Detect. Assoc. Equip..

[8] ECFA Detector R&D Roadmap Process Group (2020). The 2021 ECFA Detector Research and Development Roadmap.

[9] Gösele U., Bluhm Y., Kästner G., Kopperschmidt P., Kräuter G., Scholz R., Schumacher A., Senz S., Tong Q.-Y., Huang L.-J. (1999). Fundamental Issues in Wafer Bonding. J. Vac. Sci. Technol. A Vac. Surf. Film..

[10] Brooks A.D., Donovan R.P., Hardesty C.A. (1972). Low-Temperature Electrostatic Silicon-to-Silicon Seals Using Sputtered Borosilicate Glass. J. Electrochem. Soc..

[11] Hanneborg A., Nese M., Ohlckers P. (1991). Silicon-to-Silicon Anodic Bonding with a Borosilicate Glass Layer. J. Micromech. Microeng..

[12] Leib J., Hansen U., Maus S., Feindt H., Hauck K., Zoschke K., Toepper M. Anodic Bonding at Low Voltage Using Microstructured Borosilicate Glass Thin-Films. Proceedings of the 3rd Electronics System Integration Technology Conference ESTC.

[13] Sassen S., Kupke W., Bauer K. (2000). Anodic Bonding of Evaporated Glass Structured with Lift-off Technology for Hermetical Sealing. Sens. Actuators A Phys..

[14] Conti L., Dumont-Fillon D., Lintel H., van Chappel E. (2018). Silicon-to-Silicon Anodic Bonding via Intermediate Borosilicate Layer for Passive Flow Control Valves. Int. J. Mech. Ind. Aerosp. Sci..

[15] Tsau C.H., Schmidt M.A., Spearing S.M. (1999). Characterization of Low Temperature, Wafer-Level Gold-Gold Thermocompression Bonds. MRS Proc..

[16] Goorsky M.S., Schjølberg-Henriksen K., Beekley B., Bai T., Mani K., Ambhore P., Bajwa A., Malik N., Iyer S.S. (2018). Characterization of Interfacial Morphology of Low Temperature, Low Pressure Au-Au Thermocompression Bonding. Jpn. J. Appl. Phys..

[17] Charlot S., Pons P., Dilhan M., Vallet I., Brida S. Hermetic Cavities Using Gold Wafer Level Thermocompression Bonding. Proceedings of the Eurosensors 2017.

[18] Blom M.T., Tas N.R., Pandraud G., Chmela E., Gardeniers J.G.E., Tijssen R., Elwenspoek M., van den Berg A. (2001). Failure Mechanisms of Pressurized Microchannels: Model and Experiments. J. Microelectromechan. Syst..

